# Vesicular translocation of PARP-1 to cytoplasm causes ADP-ribosylation and disassembly of vimentin filaments during microglia activation induced by LPS

**DOI:** 10.3389/fncel.2024.1363154

**Published:** 2024-03-25

**Authors:** Ruiqi Chen, Lirui Xie, Yang Fan, Xiangmei Hua, Chang Y. Chung

**Affiliations:** ^1^School of Pharmaceutical Science and Technology, Tianjin University, Tianjin, China; ^2^Department of Biomedical Sciences, Mercer University School of Medicine, Columbus, GA, United States

**Keywords:** microglia, vimentin, PARP-1, ADP-ribosylation, ERK1/2

## Abstract

ADP-ribosylation plays a significant role in various biological processes including genomic stability maintenance, transcriptional regulation, energy metabolism, and cell death. Using macrodomain pull-down assay with microglia lysates and MALDI-TOF-MS analysis, we identified vimentin as a major protein highly ADP-ribosylated by the poly(ADP-ribose) polymerases-1 (PARP-1) in response to LPS. ABT-888, a potent inhibitor of PARP-1/2 blocks the disassembly and ADP-ribosylation of vimentin. PARP-1 is a highly abundant nuclear protein. Its nuclear functions in repairing DNA damages induced by various stress signals, such as inflammatory stresses, have been well studied. In contrast, limited studies have been done on the cytoplasmic role(s) of PARP-1. Our study focuses on the cytoplasmic role of PARP-1 during microglia activation. Using immunofluorescence microscopy and Western blotting, we showed that a significant amount of PARP-1 is present in the cytosol of microglia cells stimulated and activated by LPS. Live cell imaging showed the translocation of nuclear PARP-1-EGFP to the cytoplasm in vesicular structures upon LPS stimulation. ABT-888 and U0126 can block this translocation. Immunofluorescence staining with various organelle marker antibodies revealed that PARP-1 vesicles show colocalization with Lamin A/C, suggesting they might be derived from the nuclear envelope through nuclear envelope budding. In conclusion, we demonstrated that PARP-1 is translocated from the nucleus to cytoplasm via vesicles upon LPS stimulation and that cytoplasmic PARP-1 causes ADP-ribosylation and disassembly of vimentin filaments during microglia activation induced by LPS.

## Introduction

Microglia are the primary innate immune cells of the brain that regulate brain development, maintenance of neuronal networks, and injury repair (Colonna and Butovsky, 2017). It plays the role of phagocytosis in the central nervous system (CNS) by eliminating cell debris, microbes, and other antigens during CNS injury. Microglia perform the phagocytosis function in two highly dynamic states. Under physiological conditions, microglia are ramified cells in a steady state, characterized by having multiple thin processes and branches. These structures sense the potential injuries in the CNS. When neuron damages are detected, microglia are stimulated to transform into the activated state and morph from a ramified to an amoeboid shape (Fan et al., 2017). Upon stimulation, activated microglia migrate to the neuron damage sites in a directional way guided by the chemo-attractant gradient. This directional motility is under the regulation of various intracellular signaling cascades. The detailed regulatory mechanism of microglia activation and morphological changes is still under study.

The poly(ADP)ribose polymerase (PARP) family of enzymes modifies proteins through a posttranslational modification (PTM) by polyADP-ribosylation using NAD^+^ as a substrate (Hassa and Hottiger, 2008; Pascal, 2018). Among the 17 PARPs, PARP-1 is the most abundant (Luo and Kraus, 2012). It is DNA dependent and activated by binding to DNA strands break (Langelier and Pascal, 2013); it generates up to 90% of cellular pADPr following genotoxic stress (Qi et al., 2019). PARP-1 has a notable activity in producing pADPr in cellular stress responses. It can respond to various stress signals initiated by oxidative, nitrosative, genotoxic, oncogenic, thermal, and inflammatory stresses (Yu et al., 2002). The regulation of cellular responses through PARP-1 varies with the type and strength of stress signals. Moderate DNA damage activates PARP-1 to synthesize pADPr for regulating the sensing and repair of DNA damage. In contrast, hyperactive PARP-1 caused by severe DNA damage can lead to intracellular NAD^+^ depletion and cell death (Kim et al., 2005). Regulation on PARP-1 activity is thereby necessary.

It has been reported that microglia activation can be partly regulated by PARP-1. The anti-inflammatory effect of PARP-1 inhibitors has been reported, and a PARP inhibitor, minocycline, has been applied to suppress microglia activation (Xu et al., 2016). The mechanism underlying the regulatory role of PARP-1 in microglia activation was reported to be correlated to the polyADP-ribosylation of transcription factors via PARP-1. PARP-1 can interact with NF-κB and act as a transcriptional coactivator, further regulating the expression and release of pro-inflammatory cytokines, including protease, iNOS, and TNF-α (Kauppinen and Swanson, 2005). It was additionally found that PARP-1 genetic deficiency or inhibition of PARP-1enzyme activity could suppress TNF-α dependent microglia activation (d’Avila et al., 2012). When stimulated with TNF-α, microglia proliferated, underwent morphological changes characteristic of activation, and killed neurons placed in coculture. The effects of TNF-α were markedly attenuated both in PARP-1^–/–^ microglia and in wt microglia treated with the PARP enzymatic inhibitor DPQ (Kauppinen et al., 2006), indicating that PARP-1 activity plays a significant role in microglia activation. However, the detailed mechanism by which PARP-1 and polyADP-ribosylation regulate microglia morphological change and activation remains unclear.

Vimentin is part of the intermediate filament system which consists of GFAP, vimentin, nestin, and synemin (Helfand et al., 2011). Some evidences indicate that vimentin played a key role in controlling microglia activation. Deletion of vimentin expression significantly impaired microglia activation in response to LPS *in vitro* and transient focal cerebral ischemia *in vivo* (Jiang et al., 2012). Expression of SpyA, an ADP-ribosyltransferase of *Streptococcus pyogenes*, in HeLa cells resulted in the collapse of the vimentin cytoskeleton. In contrast, expression of SpyA in macrophage impeded vimentin reorganization upon stimulation of RAW 264.7 cells with LPS (Icenogle et al., 2012). In this study, we identified the most probable mechanism by which LPS induces the disassembly of vimentin filaments via ADP-ribosylation of vimentin by PARP-1 translocated from the nucleus to cytosol, presumably via nuclear envelope budding.

## Materials and methods

### Cell culture

BV_2_ microglia cells were cultured in Dulbecco’s Modified Eagle’s Medium (DMEM) medium (Corning, Shanghai, China) containing 10% fetal bovine serum (Biological Industries) and 100 U/ml penicillin/streptomycin (SolarBio, Beijing, China) in thermostatic incubator with 5% CO_2_. Primary microglial cells were prepared from brains of newborn pups of C57BL/6 mice (Huafukang Animal Center). Briefly, Brains were scooped out of decapitated heads. Meninges were removed, and the brain was then minced into small pieces. Tissues were dissociated into smaller pieces with repeated pipetting, and cells were collected by centrifugation, and the pellet was resuspended in DMEM with 10% fetal bovine serum and 100 U/ml penicillin/streptomycin. The cell suspension was added to poly-D-lysine (Sigma-Aldrich, Shanghai, China) coated flasks. Mixed glial cell cultures were incubated for 2 weeks, and microglia cells were detached by shaking the flasks on an orbital shaker at 200 rpm for 2 h at 37°C, and primary microglia cells were collected by centrifugation. All experiments using mice were in accordance with the Guide for the Care and Use of Laboratory Animals and were approved by the Research Ethics Committee, Tianjin Medical University.

### Western blotting

Primary or BV_2_ microglia cells (80% confluent) were used to perform experiments. For activated microglia, cells were incubated with 50 ng/ml LPS for 6 or 12 h. Cells were treated with 4 μM ABT-888 (Enzo Life Sciences) and 2 μM U0126 (SolarBio, Beijing, China) for 20 min before LPS stimulation. Cells were lysed with TBS containing 0.02% Triton X-100 (for Cytosol/Nuclear fractionation) or RIPA buffer containing protease inhibitor cocktail (Sigma-Aldrich, Shanghai, China) and phosphatase inhibitor (β-glycerophosphate; SolarBio, Beijing, China). SDS-PAGE sample buffer (5×) was added to lysates and samples were denatured by heating at 95°C. Proteins were separated by SDS-PAGE and transferred onto PVDF membrane (Millipore, Shanghai, China). The blots were blocked with 5% non-fat milk or 5% BSA in TTBS (Tris-buffered saline with 0.1% Tween-20) for 30 min and incubated overnight at 4°C with a primary antibody. Western blots were imaged with an Amersham Imager 600 (GE Healthcare), and bands were quantified using ImageJ software.

### Transfection and immunofluorescence stainings

Primary or BV_2_ microglia cells were grown on a flamed cover glass coated with poly-D-lysine at 50%–60% confluence and transfected with PARP-1-GFP (2 μg) using 8 μl of ViaFect Transfection Reagent (Promega, Beijing, China) in OPTI-MEM (Thermo Fisher, Shanghai, China) for 12 h and cells were further incubated with DMEM with 10% FBS for 12 h. Cells were then treated with LPS (50 ng; SolarBio, Beijing, China) and inhibitors for a period of time, washed with PBS, and fixed with 4% fresh formaldehyde in PBS. Cells were rinsed three times (5 min each) with TBS-Tween, and incubated with a blocking solution (5% BSA in TBS-T) for an hour. Cells were then incubated with vimentin (#5741), PARP-1 (#9542), Rab11 (#5589), Rab7 (#9367), protein disulfide isomerase (PDI; #3501), receptor binding cancer antigen expressed on SiSo cells (RCAS1; #12290), lysosome-associated membrane protein 1 (LAMP1; #51774) primary antibodies (Cell Signaling Technology, Danvers, MA, USA), or Lamin A/C primary antibody (Santa Cruz Biotechnology) overnight at 4°C and washed three times 5 min each with TBS-T. Cells were incubated with Alexa 488-secondary or Cy3-secondary antibody (Abcam), and washed three times with TBS-T. The cover glass was mounted on slides. Cells were observed under a A1 confocal Nikon or Ti fluorescence microscopes with oil-immersion 60× objective and images were taken with Qclick CCD camera (Qimaging, Tucson, AZ, USA). The length and area of cells were measured by using ImageJ software.

### Live cell fluorescence microscopy

Bv2 microglia transfected with PARP-1-GFP construct were attached to 35-mm glass-bottom dishes coated with poly-D-lysine (SolarBio, Beijing, China). When cells reached 60%–80% confluency, the media was changed to serum-free DMEM and LPS was added. Dishes were transferred to a heated (37°C) chamber and imaged using a Nikon A1 laser scanning confocal microscope with a 60× oil-immersion objective. Cells were monitored over a 12-h period by capturing images every hour. Acquisition of images was performed using Metamorph.

### GST-Af1521 pull-down assay

Af1521 is a thermophilic protein from *Archaeoglobus fulgidus*, and contains a conserved ∼190 amino acid domain known as the macrodomain. Purified macrodomain from Af1521 has been shown to bind to a subset of polymeric ADP-ribose-modified proteins with high specificity and affinity. The Af1521 macrodomain also binds to a subset of mono ADP-ribose-modified proteins. GST-Af1521 and GST-Af1521/G42E mutant (a site-specific mutant that cannot bind as a control) were expressed in BL21(DE3) bacteria using IPTG (100 mM; Sigma-Aldrich, Shanghai, China) induction. Bacterial lysates were made by sonication in lysis buffer (25 mm Tris, pH 7.6, 100 mm NaCl, 1 mm EDTA, 1% Nonidet P-40, and 1 mM dithiothreitol) containing protease inhibitor cocktail (Sigma-Aldrich, Shanghai, China). After removing cell debris by centrifugation, Glutathione-agarose beads (GenScript, Nanjing, China) were added to the supernatant of lysates and incubated for 1 h at 4°C. The beads were washed three times in lysis buffer, suspended in sample buffer, and subjected to SDS-PAGE followed by Western blotting with poly/mono ADP-ribose antibody and vimentin antibody (Cell Signaling Technology, Danvers, MA, USA). The intensity of bands was quantified by ImageJ software.

### Data analysis

All data was analyzed using Prism software (GraphPad software). Experiments were analyzed using one-way ANOVA and Tukey corrections for multiple testing between categories (indicated in figure legends). Statistical results are presented in the figure legends. The reported data for imaging studies originates from the initial analysis by the first experimenter. Data is presented with means ± standard error of the mean (SEM) shown as line and whiskers.

## Results

### ADP-ribosylation and disassembly of vimentin in response to LPS

**FIGURE 1:**
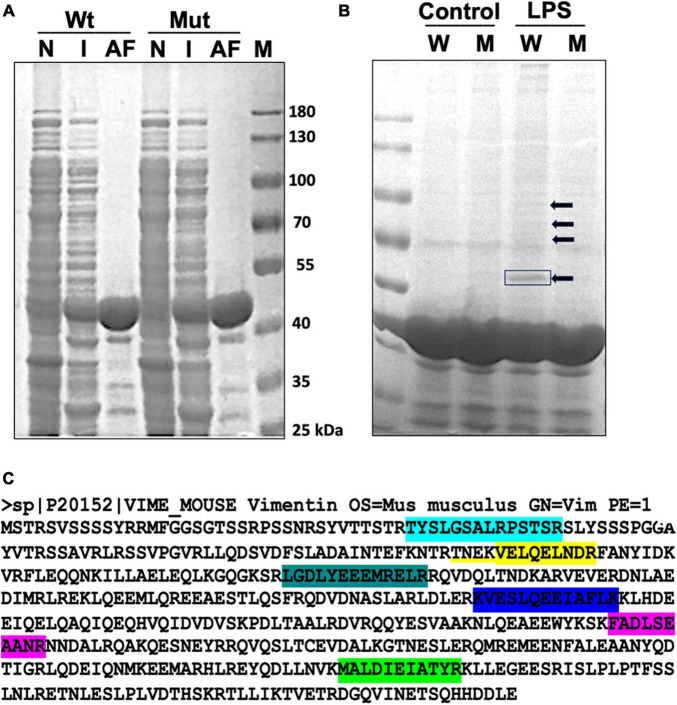
GST-Af1521 macro module pull-down of ADP-ribosylated proteins. **(A)** Expression and purification of GST-Af1521 macro module from *Escherichia coli* BL21(DE3). Non-induced bacterial lysate (N), IPTG-induced (I), and GST-Af1521 bound to glutathione beads were run on SDS-PAGE. **(B)** Af1521 macro module pull-down of ADP-ribosylated proteins from microglia lysates. Cells were untreated (Control) or treated with LPS (50 ng), and their lysates were incubated with GST-Af1521 (W) or Af1521/G42E mutant (M: a site-specific mutant that cannot bind to ADP-ribose) beads in RIPA buffer. The pulled-down samples were analyzed by SDS/PAGE and visualized by silver staining. The indicated band at 55 kDa was identified by MALDI-TOF-MS analysis. **(C)** Identification of vimentin as a major ADP-ribosylated protein upon LPS stimulation using MALDI-TOF-MS analysis. Seven matched peptides were identified.

To examine the ADP-ribosylation status of microglial proteins upon LPS stimulation, we utilized the highly selective ADP-ribose binding capacity of Af1521 in a pull-down assay. Af1521 is a protein from *A. fulgidus* and contains a conserved ∼190 amino acid domain known as the macrodomain. Purified macrodomain from Af1521 has been shown to bind to a subset of polymeric ADP-ribose-modified proteins with high specificity and affinity (Dani et al., 2009). The Af1521 macrodomain also binds to a subset of mono ADP-ribosylated proteins. We expressed and purified GST-Af1521 and GST-Af1521/G42E mutant (a site-specific mutant that cannot bind to ADP-ribose; used for detecting non-specific binding) from *Escherichia coli* BL21(DE3) and prepared glutathione-agarose beads bound to GST-Af1521 for a pull-down assay (Figure 1A). Postnuclear and cytosolic fraction isolated from Bv2 microglia cells either untreated (control) or treated with LPS were incubated with the GST-AF1521 beads, and after bead washes, bound fraction was analyzed by SDS-PAGE (Figure 1B). Multiple bands appeared on the gel from Af1521 pull-down, but neither from pull-down with control lysate or from Af1521 mutant beads. A prominent band around 55 kDa was detected and identified by MALDI-TOF-MS analysis as Vimentin. As shown in Figure 1C, seven matched peptides were identified. This result indicates that vimentin gets ADP-ribosylated in response to LPS.

**FIGURE 2:**
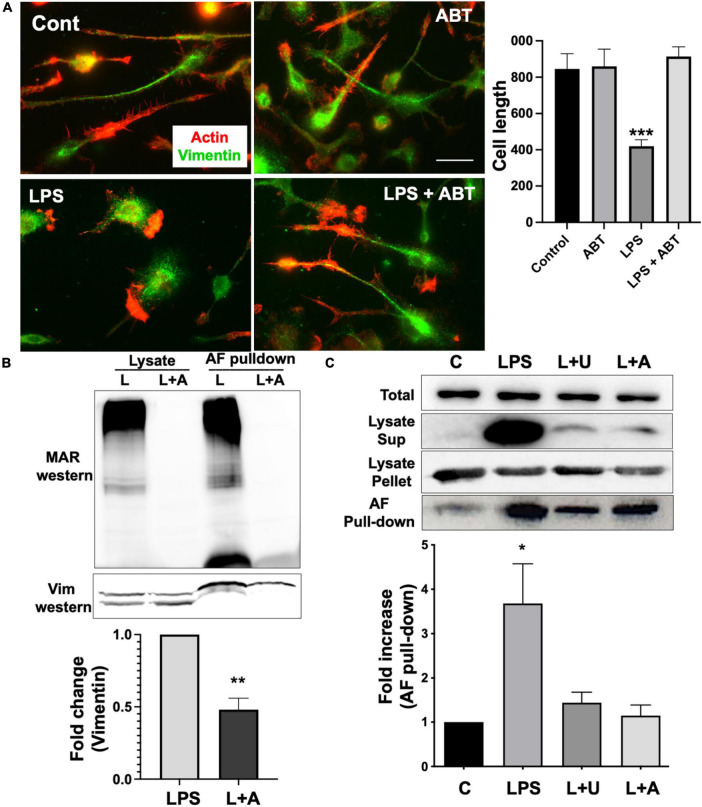
ADP-ribosylation and disassembly of vimentin in response to LPS. **(A)** Immunofluorescence staining with vimentin antibody (green) and phalloidin (red) showed the disassembly of vimentin filaments and the change of morphology of primary microglia to amoeboid shape upon LPS stimulation. Inhibition of PARP-1 with ABT-888 (L + A) blocked this transition to amoeboid shape and vimentin disassembly. Cell length was measured using line tool of ImageJ and shown in the graph. Scale bar: 10 μm. (ANOVA: ***P < 0.005 compared to control.) **(B)** Lysates of microglia treated with LPS alone or LPS with ABT-888 and the pulled-down samples with GST-Af1521 beads from these lysates were run on SDS-PAGE and probed with poly/mono-ADP ribose antibody and vimentin antibody in Western blot analysis. A significantly lower level of vimentin was detected in the pull-down from the lysate of cells treated with LPS and ABT-888, indicating the increase of vimentin ADP-ribosylation in response to LPS. Band intensity was measured using ImageJ and showed in the graph. (ANOVA: **P < 0.01 compared to control.) **(C)** Western blots of vimentin in cytosol and cytoskeleton fractions. Cell lysates were spun at 15,000 g to separate cytosolic (sup) fraction from cytoskeleton/nucleus (pellet), and vimentin was detected with Western blots. A very high level of vimentin was detected in the cytosolic fraction and AF pulled-down sample upon LPS treatment, indicating ADP-ribosylation and vimentin disassembly, which 2 μM U0126 and 4 μM ABT-888 effectively blocked. Band intensity was measured using ImageJ and showed in the graph. (ANOVA: *P < 0.05 compared to control.)

Vimentin is a part of the intermediate filament system, which plays an important physiological role in maintaining cell shape, and vimentin has been reported to function as a key controller for microglia activation (Jiang et al., 2012). Vimentin has been identified as an unusual target for ADP-ribosylation (Icenogle et al., 2012). ADP-ribosylation of vimentin occurs in an important regulatory region of the head domain, leading to the disassembly of vimentin filaments. In our previous study, we reported that vimentin filaments were observed to run parallel to the longitudinal axis of the elongated microglia, and activation of microglia by LPS induced the loss of vimentin filaments and round morphology (Fan et al., 2017). We examined if ABT-888, a potent inhibitor of PARP-1 and PARP-2 (Donawho et al., 2007), can block the disassembly of vimentin filaments. Compared to the round morphology and vimentin disassembly of LPS-treated cells, microglia treated with LPS and ABT-888 retained vimentin filaments and elongated morphology (Figure 2A), indicating ADP-ribosylation plays a significant role in vimentin disassembly. Western blot of the lysate of LPS-treated cells with an antibody recognizing poly/mono-ADP ribosyl residues showed many ADP-ribosylated proteins, and ABT-888 effectively blocked the ADP-ribosylation. ADP-ribosylated proteins were pulled down from lysates using GST-Af1521 beads and run on a SDS-PAGE gel, followed by a Western blot probed with a vimentin antibody. A significantly lower level of vimentin was detected in the pull-down from the lysate of cells treated with LPS and ABT-888 (Figure 2B), indicating a low level of ADP-ribosylation of vimentin. We then examined the amount of vimentin in the cytosol (disassembled) or cytoskeletal/nuclear (assembled) fractions using Western blots. Upon LPS treatment, a significantly higher level of vimentin was detected in the cytosol fraction but less in the cytoskeletal fraction, indicating the disassembly of vimentin filaments caused by ADP-ribosylation shown in the Western blot of Af1521 pull-down proteins. ABT-888 effectively blocked the disassembly of vimentin and also by U0126, an inhibitor of MEK (Figure 2C).

**FIGURE 3:**
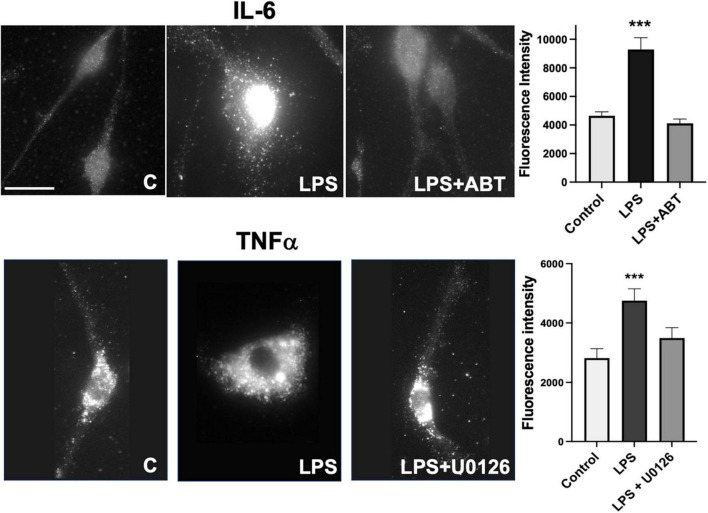
Expression of TNF-α and IL-6 in response to LPS were blocked by ABT-888 and U0126. Primary microglia were treated with LPS alone and LPS plus ABT-888 or U0126 and stained with anti-TNF-α or anti-IL-6 antibodies. Change of cell morphology to amoeboid shape and high expression of TNF-α and IL-6 (shown as many vesicles containing TNF-α and IL-6) in response to LPS were observed, which was blocked by ABT-888 and U0126. Fluorescence intensity in the cell was measured using ImageJ and showed in the graph. Scale bar: 10 μm. (ANOVA: ***P < 0.005 compared to control.)

Microglia express TNFα, IL-1β, and IL-6 in response to LPS and U0126 inhibited the expression of TNF-α and IL-1β (Ishijima and Nakajima, 2021). Suppression of vimentin expression led to up-regulation of pro-inflammatory cytokines (IL-6 and TNF-α) release but down-regulation of anti-inflammatory cytokine (IL-10) (Su et al., 2019). These findings suggested that vimentin plays an important role in the immunomodulatory mechanism. We examined if inhibiting vimentin disassembly by blocking PARP-1 activity impacts the expression of TNFα. As shown in Figure 3, microglia acquire round morphology and high expression of TNFα in response to LPS. However, the inhibition of PARP-1 activity by ABT-888 effectively restored elongated morphology and blocked the expression of TNFα. Similarly, U0126, blocking the ADP-ribosylation of vimentin, inhibited the transition to round morphology and the expression of IL-6. These results indicates an important causal link between vimentin disassembly by ADP-ribosylation and microglia activation.

### Increased cytoplasmic PARP-1 localization in microglia in response to LPS

**FIGURE 4:**
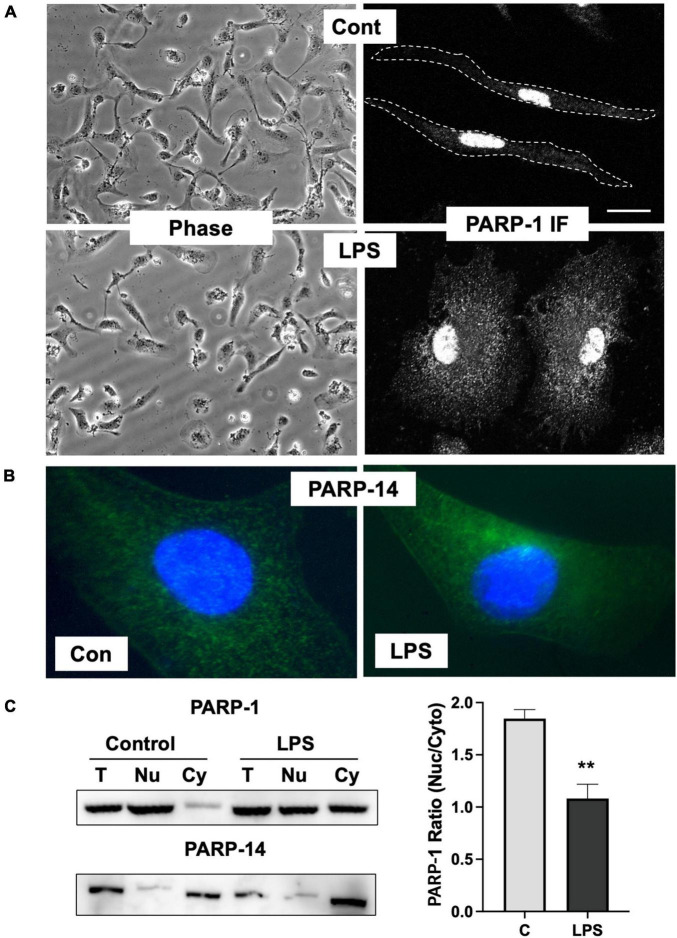
Change of PARP-1 localization in response to LPS. **(A)** Immunofluorescence staining of PARP-1 shows exclusive localization in the nucleus in untreated control cells. Upon LPS stimulation for 12 h, high fluorescence intensity from PARP-1 staining was found in the cytosol. **(B)** Immunofluorescence staining of PARP-14 shows exclusive localization in cytosol either in control or LPS-treated cells. **(C)** Cell lysates were spun at 1,300 g to separate cytosolic (Cy) fraction from nucleus (Nu) fraction, and PARP-1 and -14 were detected using Western blot. LPS treatment significantly increased vimentin in cytosol fraction, whereas PARP-14 remains mostly in cytosol. Band intensity was measured using ImageJ and the ratio between cytoplasmic to nuclear PARP-1 was showed in the graph. Scale bar: 10 μm. (ANOVA: **P < 0.01 compared to control.)

Inhibition of ADP-ribosylation of vimentin upon LPS stimulation by ABT-888 indicates that PARP-1 or 2 is the key enzyme responsible for ADP-ribosylation of vimentin. PARP-1 and 2 have been shown to be mainly localized to the nucleoplasm in addition to the nucleoli, suggesting that the subcellular localization of PARP-1 might be changed in microglia activated by LPS. To further evaluate the localization of PARP-1, primary microglia cells were stimulated with LPS, and PARP-1 localization was examined by immunofluorescence staining. Upon LPS stimulation, about 60% of cells showed round and flattened morphology. Immunofluorescence staining of PARP-1 showed an exclusive localization in nuclei, but not cytoplasm, in resting primary microglia cells. However, significant cytoplasmic localization of PARP-1 was observed in LPS-treated cells (Figure 4A). In contrast, we found that PARP-14, a mainly cytoplasmic PARP (Vyas et al., 2013), primarily localized in the cytoplasm and showed no changes of localization upon LPS stimulation (Figure 4B). To confirm the translocation of PARP-1 from the nucleus to cytoplasm, cell lysates were fractionated into cytosol and nuclear fractions, and the amount of PARP-1 in each fraction was determined by Western blot (Figure 4C). Cytoplasmic fraction of control cells exhibited a minimal level of PARP-1, but a significantly higher level of PARP-1 was detected in the cytoplasmic fraction of LPS-treated cells (Figure 4C). A high level of PARP-14 was also found in the cytoplasmic fraction of cell lysates both in control and LPS-treated microglia, indicating the dynamic change of localization upon LPS activation is specific for PARP-1.

**FIGURE 5:**
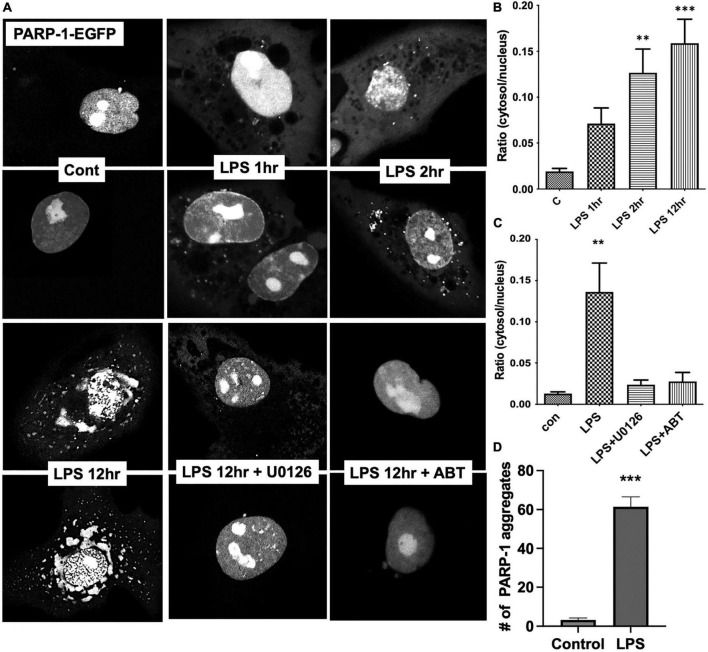
Live cell imaging showing nucleocytoplasmic translocation of PARP-1-EGFP. **(A)** Bv2 microglia were transfected with the PARP-1-EGFP construct. After LPS treatment, cells were imaged over time. Fluorescence intensity from cytoplasmic and nuclear were measured using ImageJ and ratio was shown in the graph. **(B)** Gradual increase in the number of vesicular structures containing PARP-1-EGFP over time was observed. Ratio of cytoplasmic to nuclear fluorescence intensity is shown. (ANOVA: **P < 0.01, ***P < 0.005 compared to control.) **(C)** ABT-888 and U0126 effectively blocked LPS-induced nucleocytoplasmic translocation of PARP-1-EGFP. (ANOVA: **P < 0.01 compared to control.) **(D)** In addition to cytoplasmic translocation, PARP-1-EGFP forms aggregates in the nucleus after 12 h of LPS treatment, which is also blocked by ABT-888 and 0126. (Unpaired *t*-test: ***P < 0.005 compared to control.)

To examine the dynamic changes in subcellular localization of PARP-1 in resting and LPS-stimulated cells, we transfected Bv2 microglia with PARP-1-EGFP construct, and cells were imaged over time after LPS stimulation. In resting cells, PARP-1-EGFP is primarily localized in the nucleus, especially in the nucleolar area (Figure 5A), and, upon LPS stimulation, it translocated to the cytoplasm in vesicular structures as soon as 1 h after LPS stimulation. The number of vesicles among cells at different time points after LPS stimulation was counted, and time-dependent accumulation of PARP-1-EGFP vesicles in the cytoplasm was observed (Figure 5A). The ratio of GFP intensity in the cytosol to nucleus was increased approximately sevenfold in 12 h after LPS stimulation (Figure 5B), which was effectively blocked by ABT-888 and U0126 (Figure 5C). This result is consistent with the causal link between ADP-ribosylation by PARP-1 and disassembly of vimentin filaments. In addition to this cytoplasmic localization, PARP-1-EGFP started forming small aggregates at 2 h after stimulation, and the number of aggregates peaked at 12 h (Figures 5A, D).

### Lamin A/C associates with cytoplasmic PARP-1 in vesicular structures

**FIGURE 6:**
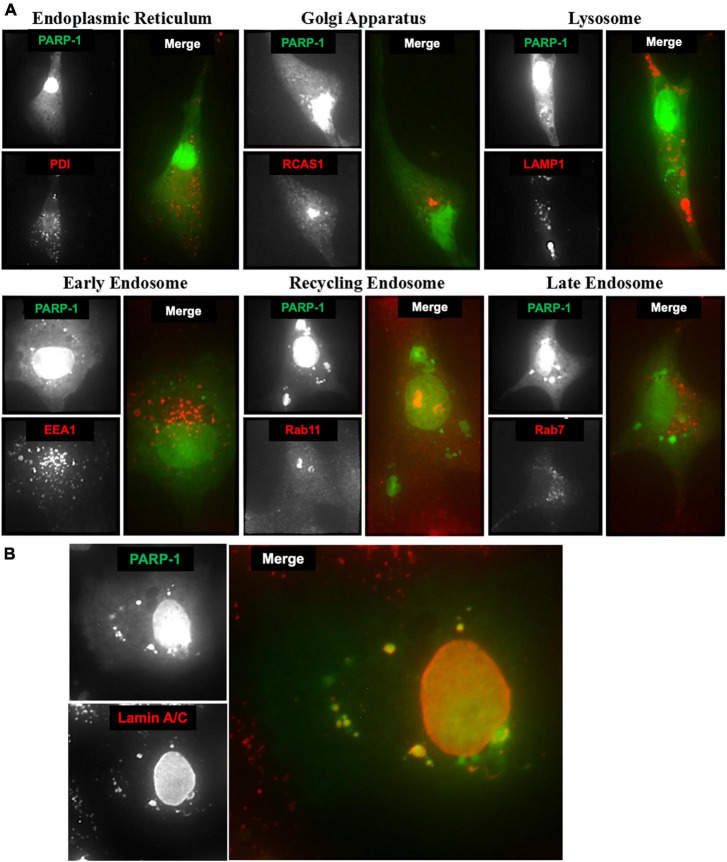
**(A)** Microglia cells expressing PARP-1-EGFP were stained with various organelle marker antibodies (red) to identify vesicular structures associated with cytoplasmic PARP-1 (green). **(B)** Lamin A/C associated with cytoplasmic PARP-1 in microglia cells expressing PARP-1-EGFP.

To identify these vesicular structures containing PARP-1, we stained microglia cells expressing PARP-1-EGFP with various organelle marker antibodies, including PDI (Yoshimori et al., 1990), RCAS-1 (Engelsberg et al., 2003), LAMP-1 (Chen et al., 1985), EEA-1 (Wurmser et al., 1999), Rab11, and Rab5 (Takahashi et al., 2012) for endoplasmic reticulum (ER), Golgi Apparatus, lysosomes, early endosomes, recycling endosomes, and late endosomes, respectively. No colocalization between cytoplasmic PARP-1 vesicular structures and PDI or RCAS1 (Figure 6A) was found, excluding the possibility that the increased amount of cytoplasmic PARP-1 was due to elevated protein synthesis or trafficking in the ER and Golgi Apparatus. The vesicular structures associated with cytoplasmic PARP-1-GFP in cells were not likely to be lysosomes or different types of endosomes since there was no colocalization either. Lack of colocalization between the PARP-1-EGFP vesicles and lysosome or endosomes and the presence of many PARP-1-EGFP vesicles in close proximity to the nucleus in Figure 4 suggests a possibility that PARP-1-EGFP vesicles might be derived from the nuclear envelope, a part of the endomembrane trafficking system. We further explored this possibility by staining microglia cells expressing PARP-1-EGFP with an antibody against Lamin A/C, a protein found in the nuclear lamina of cells, a scaffold apposed to the inner nuclear membrane (Dechat et al., 2010). The staining showed a high level of colocalization between Lamin A/C and the vesicles (Figure 6B). Some of these vesicles were distributed in the perinuclear region and formed a ring-like structure in the cytoplasm, suggesting a regulatory mechanism of PARP-1 localization via vesicular nucleo-cytoplasmic transport.

### PARP-1 translocation to cytoplasm requires its catalytic activity

**FIGURE 7:**
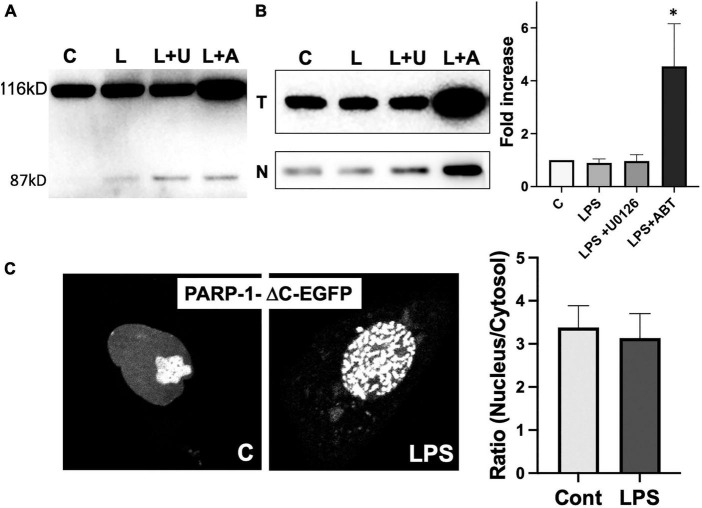
Poly(ADP-ribose) polymerases-1 (PARP-1) translocation to cytoplasm requires its catalytic activity. **(A)** PARP-1 Western blots showed minimal cleavage upon treatment of LPS and inhibitors. **(B)** A high level of PARP-1 in both total (T) lysate and nuclear fraction (N) of cells treated with LPS and ABT-888 was detected by PARP-1 Western blot. (ANOVA: *P < 0.05 compared to control.) **(C)** PARP-1-ΔC-EGFP, a mutant lacking the catalytic domain, was transfected into microglia, and its localization was examined by live cell imaging. No significant translocation of PARP-1-ΔC-EGFP to cytosol was observed in response to LPS. In contrast, the formation of PARP-1-ΔC-EGFP was unaffected.

PARP-1 is a substrate of activated caspases 3 and 7 in caspase-dependent apoptosis. The cleavage of PARP-1 by caspases 3 and 7 results in the formation of 2 specific fragments: an 89-kD catalytic fragment and a 24-kD DBD (Lazebnik et al., 1994). The 89-kD fragment containing AMD and the catalytic domain of the enzyme has a significantly reduced DNA binding capacity and is liberated from the nucleus into the cytosol (Soldani et al., 2001). To exclude the possibility that the translocation of PARP-1-EGFP to cytosol is due to the cleavage of PARP-1, we ran Western blots to examine if PARP-1 gets cleaved in response to LPS. We did not observe a significant PARP-1 cleavage in microglia cells (Figure 7A).

Inhibition of PARP-1-GFP translocation to cytoplasm by ABT-888 suggests that the catalytic activity of PARP-1 is required for the translocation. PARP-1 Western blots of total lysates and nuclear fraction of microglia showed that, in the nuclear fraction of microglia treated with LPS and ABT-888, PARP-1 expression is much higher, and a significantly larger portion of PARP-1 remains the nuclear fraction (Figure 7B). We then further examined changes of dynamic localization of PARP-1 mutant lacking the catalytic domain (PARP-1-ΔC-EGFP). PARP-1-ΔC-EGFP showed nuclear localization with a high concentration in the nucleolus and forms aggregates in response to LPS, similar to PARP-1-EGFP (Figure 7C). However, nucleo-cytoplasmic translocation of PARP-1-ΔC-EGFP was significantly lower as nuclear/cytoplasmic ratio did not show a significant change.

## Discussion

Our present study uncovered a new regulatory mechanism of PARP-1 localization between the nucleus and cytoplasm in microglia and a possible PARP-1 translocation mechanism via the formation of vesicles derived from the nuclear envelope. We also found a novel function of cytoplasmic PARP-1 in regulating vimentin filament disassembly via ADP-ribosylation of vimentin in response to LPS. This new cytoplasmic function is distinct from the known nuclear localization of PARP-1 and its nuclear function of DNA repair.

Poly(ADP-ribose) polymerases-1 is a predominantly nuclear enzyme due to its DNA damage-dependent activation pathway; most present studies focus on the functions of nuclear PARP-1, including chromatin organization, DNA damage repair, and regulation of gene transcription (Cookson et al., 1999). Whether PARP-1 can also localize in other sub-cellular structures has long been questioned. The cytoplasmic localization of PARP-1 has been reported in cancer cells (von Minckwitz et al., 2011; Xu et al., 2019). A recent study uncovered the cytoplasmic localization and function of PARP-1 in regulating DR5-activated extrinsic apoptosis pathways in pancreatic cancer cells (Rossi et al., 2009). PARP-1 expression and cellular localization are regulated and help maintain the mitochondria’s DNA integrity (Wolf et al., 2017). Other functions of cytoplasmic PARP-1 and how its subcellular localization is regulated remain unclear.

The regulatory mechanism of PARP-1 activity has been intensively studied. Different categories of posttranslational modifications, including phosphorylations on the PARP-1 modification domain, have been reported (Cohen-Armon, 2007). The significant role of ERK1/2 phosphorylation on Ser^372^ in PARP-1 enzymatic activity acquisition is widely accepted (Kauppinen et al., 2006), and PARP-1 activation by ERK1/2 is independent of DNA damage (Cohen-Armon et al., 2007). PARP-1 has been shown to be intensively activated and polyADP-ribosylated by direct interaction with phosphorylated ERK2. ERK inhibitor blocked the interaction of PARP-1 with ERK1/2, phosphorylation of PARP-1, and poly(ADP-ribosyl)ation of p65, suggesting that ERK-dependent phosphorylation of PARP-1 regulates PARP-1 activity and NF-B activation (Liu et al., 2012). Our study further demonstrated that ERK1/2 activity is also required for cytoplasmic PARP-1 localization, suggesting the role of ERK in PARP-1 translocation. A previous study showed that DNA damage induces phosphorylation on PARP-1 Thr^594^, and this leads to the cytosolic translocation of PARP-1 (Wang et al., 2022). Mechanistic details of the cytosolic translocation of PARP-1 need to be unveiled.

Vesicular nucleo-cytoplasmic transport is becoming recognized as a general cellular mechanism for translocating large cargoes across the nuclear envelope (Hagen et al., 2015). Cargo is recruited, enveloped at the inner nuclear membrane (INM), and delivered by membrane fusion at the outer nuclear membrane. In this study, we found an elevated cytoplasm-to-nucleus PARP-1 ratio in response to LPS, and immunofluorescence microscopy showed colocalization of PARP-1-GFP vesicular structures with nuclear envelope stained with Lamin A/C antibody. This association suggests possible nucleocytoplasmic trafficking of PARP-1 via nuclear envelope budding (NEB). NEB pathway was previously reported to be exclusive to the nuclear egress of Herpes viruses (HVs) due to the large size of HV nucleocapsids (∼ 125 nm) limited by the diameter of the central channel of nuclear pore complex (NPC) (Fradkin and Budnik, 2016). Later, an endogenous cellular NEB pathway similar to HV nuclear egress was reported to play a role in the exit of large ribonucleoproteins, Fz2C RNPs (Speese et al., 2012). It has recently been shown that NEB is a ubiquitous eukaryotic phenomenon and increases when exposed to various forms of cellular stress (Panagaki et al., 2021). NEB events might be a more efficient gateway out of the nucleus for the transportation of many large protein aggregates. Our study demonstrated the formation of PARP-1-GFP aggregates in the nucleus upon LPS stimulation, which is consistent with the idea of NEB being the primary way PARP-1 is transported out of the nucleus. Further study on mechanistic details is needed to evaluate whether NEB is the primary way of PARP-1 translocation.

Many pieces of evidence indicate that the nuclear lamins are connected to the cytoskeleton through a complex of proteins called the LINC complex (linker of nucleoskeleton and cytoskeleton) (Dechat et al., 2010). Studies demonstrated the functional significance of this interaction between the nuclear lamina and the cytoskeleton. Lamin A-deficient fibroblasts are reported to show defects in cell polarization and cell migration and a disturbed organization of vimentin IF networks (Houben et al., 2009). The dynamic variations in lamin A/C and vimentin expression establish a positive feedback loop in response to confinement, effectively promoting amoeboid migration by modulating nuclear deformability while ensuring cell viability (Wang et al., 2023). Our study demonstrated the disassembly of vimentin filaments resulting from ADP-ribosylation in response to LPS, which might have some impacts on the nuclear lamin network and nuclear envelope budding.

## Data availability statement

The original contributions presented in this study are included in this article/supplementary material, further inquiries can be directed to the corresponding author.

## Ethics statement

The animal study was approved by the Research Ethics Committee, Tianjin Medical University. The study was conducted in accordance with the local legislation and institutional requirements.

## Author contributions

RC: Conceptualization, Data curation, Formal analysis, Investigation, Writing - original draft, Writing – review & editing. LX: Writing – original draft, Writing – review & editing. YF: Conceptualization, Data curation, Formal analysis, Investigation, Writing – original draft, Writing – review & editing. XH: Data curation, Formal analysis, Investigation, Writing – original draft, Writing – review & editing. CC: Conceptualization, Data curation, Formal analysis, Funding acquisition, Investigation, Methodology, Project administration, Resources, Software, Supervision, Validation, Visualization, Writing – original draft, Writing – review & editing.
